# Spatially-explicit risk profiling of *Plasmodium falciparum *infections at a small scale: a geostatistical modelling approach

**DOI:** 10.1186/1475-2875-7-111

**Published:** 2008-06-23

**Authors:** Kigbafori D Silué, Giovanna Raso, Ahoua Yapi, Penelope Vounatsou, Marcel Tanner, Eliézer K N'Goran, Jürg Utzinger

**Affiliations:** 1 UFR Biosciences, Université de Cocody-Abidjan, 22 BP 770, Abidjan 22, Côte d'Ivoire; 2 Centre Suisse de Recherches Scientifiques, 01 BP 1303, Abidjan 01, Côte d'Ivoire; 3 School of Population Health, University of Queensland, Herston Road, Brisbane, QLD 4006, Australia; 4 Molecular Parasitology Laboratory, Queensland Institute of Medical Research, 300 Herston Road, Brisbane, QLD 4006, Australia; 5 Department of Public Health and Epidemiology, Swiss Tropical Institute, P.O. Box, CH-4002 Basel, Switzerland

## Abstract

**Background:**

There is a renewed political will and financial support to eradicate malaria. Spatially-explicit risk profiling will play an important role in this endeavour. Patterns of *Plasmodium falciparum *infection prevalence were examined among schoolchildren in a highly malaria-endemic area.

**Methods:**

A questionnaire was administered and finger prick blood samples collected from 3,962 children, aged six to 16 years, attending 55 schools in a rural part of western Côte d'Ivoire. Information was gathered from the questionnaire on children's socioeconomic status and the use of bed nets for the prevention of malaria. Blood samples were processed with standardized, quality-controlled methods for diagnosis of *Plasmodium *spp. infections. Environmental data were obtained from satellite images and digitized maps. Bayesian variogram models for spatially-explicit risk modelling of *P. falciparum *infection prevalence were employed, assuming for stationary and non-stationary spatial processes.

**Findings:**

The overall prevalence of *P. falciparum *infection was 64.9%, ranging between 34.0% and 91.9% at the unit of the school. Risk factors for a *P. falciparum *infection included age, socioeconomic status, not sleeping under a bed net, distance to health care facilities and a number of environmental features (i.e. normalized difference vegetation index, rainfall and distance to rivers). After taking into account spatial correlation only age remained significant. Non-stationary models performed better than stationary models.

**Conclusion:**

Spatial risk profiling of *P. falciparum *prevalence data provides a useful tool for targeting malaria control intervention, and hence will play a role in the quest of local elimination and ultimate eradication of the disease.

## Background

Malaria remains a leading cause of morbidity and mortality in tropical and subtropical regions of the world. There are an estimated three billion people at risk of this disease and more than half a billion episodes of clinical *Plasmodium falciparum *occur each year, killing over one million individuals annually [1,2]. It has been estimated that the global burden of malaria exceeds 40 million disability-adjusted life years (DALYs) [2,3] and the disease drains the social and economic development of affected regions [4,5]. High-risk groups are children under the age of five years and pregnant women, with sub-Saharan Africa particularly affected. Indeed, this part of the world accounts for a striking 90% of the global burden of malaria [6,7].

Predicting the abundance and spread of malaria in endemic settings, in order to develop locally-adopted malaria control strategies to lower the burden of the disease is a pressing public health issue. Recently, an audacious goal has been announced in Seattle, USA during a meeting led by the Bill and Melinda Gates Foundation, namely to eradicate malaria [8]. This goal – so the claim – has become a realistic hope thanks to new scientific advances, including the development of novel antimalarial drugs, vaccines and integrated control efforts through insecticide-treated nets (ITNs), prophylactic treatment and indoor residual spraying (IRS), in the face of a growing political will and financial support for malaria control initiatives [8]. A deeper understanding of the spatial distribution of malaria is pivotal so that appropriate local elimination efforts can be designed and rigorous monitoring implemented.

Advances made with geographical information system (GIS), remote sensing and geostatistical modelling to predict the spatial and temporal distribution of malaria and *Anopheles *vectors have opened new avenues in this field of research. In particular, modelling disease and disease-related data within a Bayesian framework allows fitting of complex models in quite a flexible way. Additionally, Bayesian approaches provide computational advantages over traditional frequentist approaches via implementation of Markov chain Monte Carlo (MCMC) simulation [9-11]. Recent studies made use of the advantages offered by Bayesian methods for spatially-explicit modelling of malaria [12-18].

In Côte d'Ivoire, malaria is one of the primary public health concerns. This is illustrated by a study carried out in the savannah zone that documented malaria being responsible for at least 60% of the consultations in hospitals and 46% in paediatric clinics [19]. In 2005, Côte d'Ivoire ranked at position 13 among countries with the highest rates of under-five mortality and estimates at the time suggested that only 4% of children under five years of age slept under an ITN [20]. In the present study, small-scale patterns and spatial risk factors of the prevalence of *P. falciparum *among schoolchildren in a rural part of western Côte d'Ivoire were explored, using Bayesian geostatistical models.

## Methods

### Study area, population and ethical clearance

The study area is the region of Man, located in western Côte d'Ivoire. It is a mountainous region with tropical climate, including rains during eight to nine months of the year, and a dry period between November and February. The landscape is characterized in the north by rounded mountains with altitudes ranging from 200 to 1,300 m above sea level and small valleys, whereas the southern part is a river-draining plain [21]. The field work was carried out between October 2001 and February 2002.

The study protocol was approved by the institutional research commission of the Swiss Tropical Institute (Basel, Switzerland) and the Centre Suisse de Recherches Scientifiques (Abidjan, Côte d'Ivoire). The study was given ethical clearance from the Ministry of Health in Côte d'Ivoire. All children attending grades three to five from 57 schools in the rural parts of the study area were invited to participate.

### School surveys

The education officers were contacted and the aims and procedures of the study were explained. After receipt of their approval, the education officers informed teachers who provided the research team with copies of the class lists, which included information of the children's name, sex and age. First, a questionnaire was administered and schoolchildren were interviewed for assets on ownership and household characteristics, and perceived symptoms and diseases with a recall period of one month. The questionnaire included 17 morbidity indicators (e.g. abdominal pain, fever, etc.) and 12 household assets (e.g. radio, TV, etc.). An asset-based approach was used to stratify schoolchildren into five socioeconomic groups [22,23]. An additional question was included to inquire whether children slept under a bed net.

Second, a cross-sectional survey was carried out, to collect finger prick blood samples from previously interviewed children. Two drops of blood were placed on a microscope slide and thin and thick blood films were prepared. Slides were air-dried, transferred to a laboratory in the town of Man and stained with Giemsa. The slides were then forwarded to a reference laboratory in Abidjan and analysed by experienced laboratory technicians for species-specific density of *Plasmodium*, assuming a standard white blood cell (WBC) count of 8,000 per μl of blood by light microscopy. A random sample of 10% of the slides were re-examined by the senior microscopist for quality control purposes. Since more than 95% of the cases were *P. falciparum *infections, subsequent spatial analyses was restricted on this malaria parasite.

### Environmental data

Geographical coordinates of each school were collected using a hand-held Magellan 320 global positioning system (GPS; Thales Navigation; Santa Clara, CA, USA). Streets and rivers were digitized with the aid of readily available ground maps. Normalized difference vegetation index (NDVI) and land surface temperature (LST) were downloaded at 1 × 1 km spatial resolution from Moderate Resolution Imaging Spectroradiometer (MODIS) from the USGS EROS Data Centre. Rainfall estimate (RFE) data with an 8 × 8 km spatial resolution from Meteosat 7 satellite were obtained from the Africa Data Dissemination Service (ADDS). NDVI, LST and RFE were downloaded for the period of September, 2001 to August, 2002 and processed as detailed elsewhere [21]. Distances from schools to the nearest healthcare facility and rivers were calculated.

### Data management and statistical analysis

Data were entered twice and validated with EpiInfo version 6.4 (Centers for Disease Control and Prevention; Atlanta, GA, USA). Geographical data were displayed in ArcView GIS version 3.2 (Environmental Systems Research Institute, Inc.; Redlands, CA, USA). Schoolchildren were subdivided into two age groups; (i) six to 10 years, and (ii) 11 to 16 years.

All covariates were fitted into bivariate logistic regression models on the *P. falciparum *infection status variable using STATA version 9.2 (Stata Corporation; College Station, TX, USA). Covariates with a significance level <0.15 were built into (i) a stationary, and (ii) a non-stationary Bayesian logistic regression model for *P. falciparum *infection, using WinBUGS version 1.4 (Imperial College & Medical Research Council; London, UK). The stationary geostatistical model assumed that spatial correlation is a function of distance only, whereas the non-stationary geostatistical model assumed that spatial correlation is a function of the distance and location [16,24]. Spatial heterogeneity was taken into account by introducing location-specific random effects, which model a latent spatial process.

### Model specification

Let *Y*_*ij *_be the *P. falciparum *infection status of schoolchild *j *in school *i*. It is assumed that *Y*_*ij *_arises from a Bernoulli distribution, *Y*_*ij *_~*Be*(*P*_*ij*_), with probability *P*_*ij*_. The covariates *X*_*ij *_and school-specific random effect *φ*_*i *_were modelled on the $\log it(p_{ij})={\underline{X}}_{ij}^{T}\underline{\beta }+\varphi_{i}$, that is log *it *(*P*_*ij*_), where *β* is the vector of regression coefficients.

The spatial correlation was introduced on the *φ*_*i*_'s by assuming that *φ* = (*φ*_1_, *φ*_1_, ... *φ*_*N*_)^*T *^has a multivariate normal distribution, *φ* ~*MVN*(0, Σ), with variance-covariance matrix Σ. An isotropic spatial process, i.e. Σ_*mn *_= *σ*^2 ^exp (-*ud*_*mn*_), was also assumed, where *d*_*mn *_is the Euclidean distance between schools *m *and *n*, *σ*^2 ^is the geographic variability known as the sill, and *u *is a smoothing parameter that controls the rate of correlation decay with increasing distance. To take into account non-stationarity, the study area was partitioned in *K *subregions, assuming a locally stationary spatial process *ω*_*k *_in each subregion *k *= 1, ..., *K*, where *ω*_*k *_= (*ω*_*k*1_, *ω*_*k*2_, ..., *ω*_*kN*_)^T^. In order to separate the schools into approximately equal numbers, the study area was subdivided into two subregions on a diagonal from the north-western corner to the south-eastern corner. Spatial correlation in the study area was then viewed as a mixture of the different spatial processes and the spatial random effect *φ*_*i *_at location *i *was modelled as a weighted average of the subregion-specific (independent) stationary processes as follows: $\varphi_{i}=\sum_{k=1}^{K}a_{ik}\omega_{ki}$, with weights *a*_*ik*_, which are decreasing functions of the distance between location *i *and the centroids of the subregions *k *[25]. Assuming *ω*_*k *_~ *MVN*(0, Σ_*k*_) and ${\left(\sum_{k}\right)}_{ij}=\sigma_{k}^{2}corr(d_{ij};u_{k})$, one has $\underline{\varphi }=N(\underline{0},\sum_{k=1}^{K}A_{k}^{T}\sum_{k}A_{k})$, where *A*_*k *_= *diag*{*a*_1*k*_, *a*_2*k*_, ..., *a*_*nk*_}. The range is defined as the minimum distance at which spatial correlation between locations is below 5%. For an exponential correlation function, it can be calculated as 3uk meters.

Following a Bayesian model specification, prior distributions were adopted for the model parameters. Vague Normal distributions for the *β* parameters with large variances (i.e. 10,000), inverse gamma priors for $\sigma_{k}^{2}$ and uniform priors for *u*_*k*_, *k *= 1, ..., *K *were chosen. MCMC simulation was employed to estimate the model parameters [26]. A single chain sampler with a burn-in of 5,000 iterations was run. Convergence was assessed by inspection of ergodic averages of selected model parameters.

### Model performance

The deviance information criterion (DIC) was utilized to assess the model performance [27]. For appraisal of the predictive ability of models, a training sample from the current database was used. From the 55 schools, 43 schools (78%) were randomly selected and fitted into the logistic regression models. The remaining 12 schools were utilized for validation purposes. 95%, 75%, 50%, 25% and 1–5% Bayesian credible intervals (BCIs) of the posterior predictive distribution of test locations were calculated. The model with the highest percentage of locations within the BCI with the smallest coverage was considered the best performing one.

## Results

### Study profile and operational results

**Figure 1:**
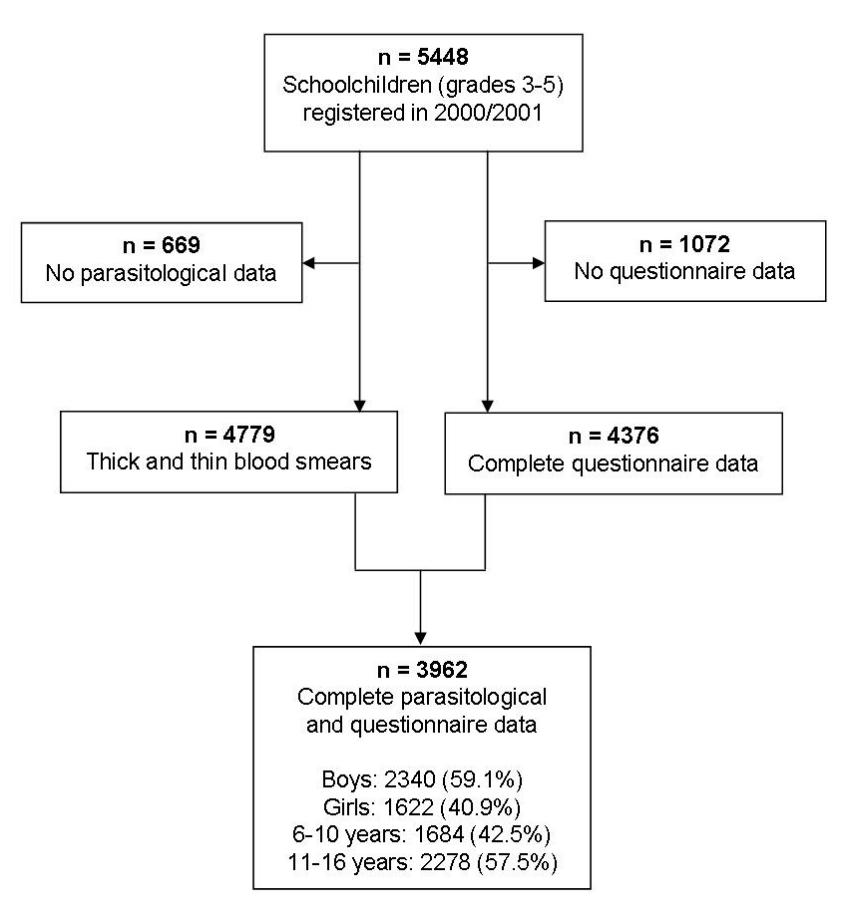
Study profile and compliance.

In the school year 2001/2002, a total of 5,448 children were registered on the class lists of grades three to five of the 57 participating rural schools. Complete questionnaire and parasitological data were obtained from 3,962 schoolchildren (72.7%) in 55 schools (one school failed to return the questionnaires; no blood samples were collected in another school). All subsequent analyses are based on this final cohort (Figure 1).

### Plasmodium infections

**Figure 2:**
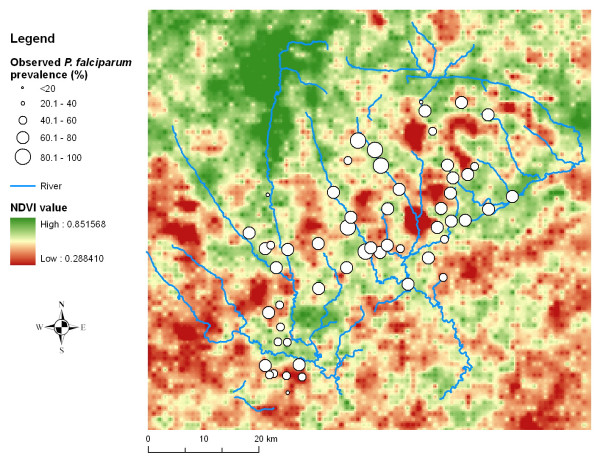
**Mean infection prevalence of *P. falciparum *in 55 rural schools in the Man region, western Côte d'Ivoire during the school year 2001/2002**. NDVI is displayed in the background.

Approximately two-thirds of the study population were infected with malaria parasites. *P. falciparum *was the predominant species (overall prevalence 64.9%), whereas infections with *Plasmodium malariae *and *Plasmodium ovale *were rare; the respective prevalences were 3.0% and 0.2%. At the unit of the school, the *P. falciparum *prevalence ranged from 34.0% to 91.9% (Figure 2).

### Risk profiling and spatial patterns

Results of the bivariate non-spatial analyses considering demographic, socioeconomic and environmental covariates, distance to health care facilities and use of bed net are summarized in Table 1. While children aged six to 10 years were at a significantly higher risk of a *P. falciparum *infection than their older peers, no difference was found among boys and girls. Children from the third wealth quintile (poor) were at a significantly higher risk of having an infection with *P. falciparum *compared to the first wealth quintile (poorest group). Besides age and socioeconomic status, not sleeping under a bed net, distance to health care facilities, and three environmental factors (i.e. high NDVI, high RFE and attending schools located at a distance to rivers of 500 m to 1000 m) were risk factors for a *P. falciparum *infection.

**Table 1:** Results of the bivariate logistic regression model for *P. falciparum *infections among 3,962 children from 55 rural schools in the Man region, western Côte d'Ivoire.

Indicators	*P. falciparum *infection prevalence		
	
	OR^a^	95% CI	*P*-value (AIC^b^)
Age (years)			
6–10	1		
11–16	0.70	0.61, 0.8	<0.001
Socioeconomic status			
Most poor	1		
Very poor	1.04	0.84, 1.28	
Poor	1.24	1.01, 1.53	
Less poor	0.95	0.77, 1.16	
Least poor	0.86	0.70, 1.06	0.011
Sleeping under a bed net	0.79	0.65, 0.98	0.030
Distance to health care facility	1.17	1.09, 1.25	<0.001
NDVI			
Mean I^c^	0.96	0.91, 1.03	0.333
Mean II^d^	1.05	0.99, 1.12	0.131 (5139)
Mean III^e^	0.96	0.90, 1.03	0.228
Annual mean	1.20	1.12, 1.29	<0.001 (5110)
Mean of the transmission season	1.19	1.11, 1.27	<0.001 (5114)
RFE			
Mean I^c^	0.88	0.82, 0.94	<0.001 (5126)
Mean II^d^	1.04	0.97, 1.11	0.289
Mean III^e^	1.13	1.05, 1.20	<0.001 (5129)
Sum of annual rainfall	1.11	1.04, 1.18	0.003 (5132)
Mean of the transmission season^f^	1.14	1.07, 1.21	<0.001 (5125)
Maximum LST	1.03	0.96, 1.09	0.449
Distance to rivers (categorized)			
<500 m	1		
500–999 m	1.22	1.03, 1.46	
= 1000 m	0.63	0.54, 0.74	<0.001

^a^OR: odds ratio

^b^AIC: Akaike information criterion. The smaller the AIC the better the model performance

^c^Mean I: Mean value during the month prior to blood sample collection

^d^Mean II: Mean value during the month of collection and the previous month

^e^Mean III: Mean value during the month of collection and the two previous months

^f^The main transmission season of malaria is from June to August

### Spatial analyses

Results of the spatial analyses are summarized in Table 2. Only age was a significant risk factor for *P. falciparum *prevalence, both in the stationary and the non-stationary logistic regression model, whereas NDVI was 'borderline' significant. In general, for all indicators, odds ratios (ORs) were comparable between the stationary and the non-stationary logistic regression model. The range where spatial correlation became insignificant was similar between the stationary and the non-stationary model. The non-stationary model revealed that location only had a minor leverage on the range of spatial correlation. The geographical variability was 0.3 with the stationary model, whereas in the non-stationary model there was some difference in the geographical variability between the two subregions.

**Table 2:** Multivariate stationary and non-stationary spatial analyses results for *P. falciparum *infection prevalence for the Man region, western Côte d'Ivoire.

Indicator	Bayesian logistic regression models			
	
	Stationary		Non-stationary	
	
	OR^a^	95% BCI^b^	OR^a^	95% BCI^b^
Age (years)				
6–10	1		1	
11–16	0.75	0.65, 0.87	0.75	0.65, 0.87
Socioeconomic status				
Most poor	1		1	
Very poor	0.90	0.71, 1.13	0.90	0.71, 1.13
Poor	1.21	0.95, 1.51	1.21	0.95, 1.51
Less poor	0.91	0.90, 1.15	0.90	0.71, 1.14
Least poor	0.85	0.66, 1.08	0.84	0.65, 1.08
Sleeping under a bed net	0.92	0.72, 1.15	0.92	0.73, 1.15
Distance to health care facility	1.07	0.87, 1.29	1.04	0.82, 1.27
Annual mean NDVI	1.16	0.98, 1.38	1.17	0.98, 1.40
Mean RFE during transmission season	1.06	0.87, 1.27	1.06	0.87, 1.27
Distance to rivers				
<500 m	1		1	
500–999 m	1.32	0.87, 1.94	1.27	0.81, 1.89
= 1000 m	0.75	0.48, 1.14	0.72	0.47, 1.09
*ρ*_1_^c^	0.0014	0.0003, 0.002	0.0015	0.0003, 0.002
*ρ*_2_			0.0014	0.0004, 0.002
*σ*_1_^2d^	0.30	0.17, 0.49	0.23	0.10, 0.48
*σ*_2_^2^			0.40	0.18, 0.79
**DIC^e^**	**4899.8**		**4900.1**	

^a^OR: odds ratio

^b^BCI: Bayesian credible interval

^c^*ρ*: scalar parameter representing the rate of decline of correlation with distance between points

^d^*σ*^2^: estimate of geographic variability

^e^DIC: deviance information criterion; a composite measure of how well the model does, i.e. a compromise between fit and complexity, with smaller DICs indicating better performance of the model

### Model performance

Comparison of DICs suggested that both the stationary and the non-stationary model were similar with regard to their performance. Since the DIC did not give any definite information on the best-fitting models, further exploration was necessary by data training. Table 3 shows the results of the model validation. The non-stationary logistic regression model predicted correctly 100% of the test locations compared to 93% with the stationary logistic regression model at a 95% BCI. Moreover, the non-stationary logistic regression model had the highest percentage of correctly predicted locations at the smallest BCI, and hence can be regarded as the best fitting model.

**Table 3:** Percentage of test locations with *P. falciparum *prevalence falling within selected BCIs. For the model validation 43 locations were used for model fitting and 12 for prediction.

BCI	Bayesian logistic regression model	
	
	Stationary	Non-stationary
95%	93%	100%
75%	80%	87%
50%	53%	60%
25%	27%	27%
5%	13%	27%
4%	7%	27%
3%	7%	27%
2%	7%	20%
1%	7%	13%

## Discussion

The purpose of this study was to assess risk factors and small-scale spatial patterns of *P. falciparum *infection prevalence among schoolchildren in a highly endemic area of rural western Côte d'Ivoire. The following covariates were significantly associated with infection: age, socioeconomic status, sleeping under a bed net, distance to health care facilities and a number of environmental factors. However, after accounting for spatial correlation, only age remained a significant risk factor for *P. falciparum *prevalence, whereas NDVI showed only 'borderline' significance. The predictive ability of the spatial models was examined using a training sample of 78% of the schools, with the non-stationary model performing better than the stationary one.

There are a number of shortcomings worth discussing. First, only a single finger prick blood sample was collected from each child for microscopic examination. Hence it is conceivable that some infections, particularly those with a low parasitaemia, were missed [28,29]. Second, it should be noted that school-aged children in highly malaria-endemic areas are not at highest risk of disease-associated morbidity and mortality. The prevalence in children below the age of five years might have been even higher than the observed *P. falciparum *prevalence of 64.9% among six to 16-year-old children. Third, the parasitological survey was carried out over a period of several months due to the large number of schoolchildren subjected to interviews and finger prick blood sampling, which might have introduced a bias in the observed prevalence from one school to another due to seasonality. Fourth, in the absence of high-resolution data to compute distances to small standing water bodies that might serve as *Anopheles *breeding sites, information from digitized maps was used to obtain the distance to rivers as an indication for the distance to breeding sites. The most likely vector in this area is *Anopheles gambiae *and, to some extent, *Anopheles funestus*. The former vector species breeds in transient, sunlit and generally small pools, whereas the latter has been associated with larger, semipermanent bodies of water containing aquatic vegetation and algae [30].

The analysis presented here showed that schoolchildren from wealthier households were more likely to be infected with *P. falciparum *compared to schoolchildren from the poorest households. This result is surprising given that the common expectation would be that the poorest of the poor are at highest risk of malaria [31]. Several studies have shown that the burden of malaria is elevated among the poorest population segments, probably because they are at a higher exposure to malaria vectors and have fewer means for personal protective measures. For example, a study carried out in a rural community in Cameroon found a significant relationship between malaria and low protective housing conditions, such as living in wooden plank houses [32]. Surprisingly, no significant association between the risk of a *P. falciparum *infection and housing conditions was evident in the present study. It is conjectured that issues related to exposure were associated to socioeconomic status, which calls for further investigation. Previous research conducted in rural Tanzania, for example, found that lack of access to health care and preventive measures, including ITNs, was associated with people's socioeconomic status [31]. Interestingly, the current study confirms that children from poorer households were less likely to sleep under a bed net. Furthermore, children who reported sleeping under a bed net were at a decreased risk of having a *P. falciparum *infection. Additionally, it was found that the risk of a *P. falciparum *infection was associated with distances to health care facilities. Nevertheless, after taking into account spatial correlation, the covariates socioeconomic status, distance to the nearest health care facility and sleeping under a bed net showed no significant association anymore, and hence other factor must explain the observed spatial heterogeneity of *P. falciparum*.

Several environmental factors, namely NDVI, RFE and distance to rivers, were significantly associated with a *P. falciparum *infection in the bivariate non-spatial models. These findings are in accordance with previous studies that showed significant associations between malaria and NDVI, rainfall and distance to rivers at a broader spatial scale [33-35]. It is conceivable that these environmental factors are related to the presence and abundance of malaria vectors, which is governed by suitable breeding and resting sites of *Anopheles*. An interesting observation in the present study was that children from schools that were located in close proximity to rivers (<500 m) were at a lower risk of a *P. falciparum *infection compared to more distant schools (between 500 m and 1000 m). Children from schools with distances <500 m were significantly more often reporting to sleep under a bed net, suggesting that the former observation might be partly confounded by a higher level of bed net coverage and usage due to nuisance from mosquitoes near rivers. Children enrolled in schools located at distances >1000 m of rivers were less likely to be infected with *P. falciparum*, which might be related to the flight range of mosquitoes, which is, on average, below 1 km [36]. Interestingly, none of the environmental covariates showed a statistical significant association to *P. falciparum *prevalence after accounting for spatial correlation. Hence, the current results demonstrate the importance of accounting for spatial correlation when analysing malaria prevalence data at small spatial scales as reported here. Indeed, omission of spatial correlation would have underestimated the standard errors of the covariate coefficients [37]. Furthermore, in contrast to previous work focussing on helminth infections in the same study area [11,21,24,38], no risk map and corresponding uncertainty map have been presented, since none of the environmental factors investigated was significant in the spatially-explicit model. The results therefore suggest that at small spatial scales, individual-level factors (e.g. age) determine the spatial distribution of the *P. falciparum *infections rather than coarser environmental factors. These observations suggest that environmental factors are particularly salient for malaria prediction at larger spatial scales.

In geostatistical modelling, the standard assumption is that there is a stationary spatial dependence in the data, which implies that the spatial correlation is a function of the distance between points and independent of the location. Bayesian non-stationary geostatistical models were employed before for the prediction of helminth infections in the same study area [24,38]. Gosoniu and colleagues were the first to use Bayesian non-stationary geostatistical models for malaria risk, in their recent research on Mali [16] and West Africa [39]. The authors' underlying assumption was that local characteristics related to human behaviour and environment, including vector ecology, influenced spatial correlation differently at different locations over large areas, i.e. an entire country. The results presented here suggest that the use of non-stationary models may also be required at a smaller spatial scale (i.e. at the district level), since the non-stationary model performed better than the one assuming stationarity. The current work on *P. falciparum *can be integrated with our previous work on helminth infections for mapping *P. falciparum*-helminth co-infections using multinomial regression models for the simultaneous targeting of malaria and helminthic diseases [11]. School-aged children are at the highest risk of such co-infections and data suggest that co-infections with *P. falciparum *and hookworm have an additive impact on anaemia, implying that those high-risk groups would greatly benefit from integrated malaria and helminth control [40].

## Conclusion

An integrated approach that employs different data sources, GIS and remote sensing technologies and Bayesian geostatistical modelling for spatially-explicit risk profiling of *P. falciparum *infection prevalence in a highly malaria-endemic part of sub-Saharan Africa was used. This approach can be readily adapted to other eco-epidemiological settings for spatial targeting of control interventions. In particular, it was possible to compare different geostatistical models with a large set of covariates, including demographic, socioeconomic and environmental factors, physical access to health care and bed net usage. The results suggest that the use of non-stationary models might be justified also at small-scale areas, however further research is necessary to deepen the current understanding of the fine-scale spatial heterogeneity of *P. falciparum*. Malaria patterns are complex and the risk of infection is influenced by many other factors that were not accounted for in this study, including malaria control interventions and genetic diversity. Specifically, vector breeding sites at small scale (i.e. abundance of small water pools) may significantly influence the spatial heterogeneity in the study area [41-43]. Further analyses that apply information derived from land use maps are needed, as well as models to predict the spatial distribution of *P. falciparum *parasitaemia.

## List of abbreviations

ADDS: Africa Data Dissemination Service; AIC: Akaike Information Criterion; BCI: Bayesian Credible Interval; CI: Confidence Interval; DALY: Disability-Adjusted Life Year; DIC: Deviance Information Criterion; GIS: Geographical Information system; GPS: Global Positioning System; IRS: Indoor Residual Spraying; ITN: Insecticide-Treated Net; LST: Land Surface Temperature; MCMC: Markov Chain Monte Carlo; MODIS: Moderate Resolution Imaging Spectroradiometer; NDVI: Normalized Difference Vegetation Index; OR: Odds Ratio; RFE: Rainfall Estimates; WBC: White Blood Cell

## Authors' contributions

KDS contributed to the conception and design of the study, collected the data, was responsible for quality control issues of malaria slide reading, assisted with the analysis of the data and editing of the manuscript, GR contributed to the conception and design of the study, collected the data, analysed and interpreted the data and drafted and edited the manuscript, AY was involved in the collection of the data and supervision of the field work, PV contributed to the analysis of the data and editing of the manuscript, MT contributed to the conception and design of the study, EKN and JU oversaw all aspects of the study, including conception, design, execution of the field work, interpretation of the data and editing of the manuscript. All authors read and approved the final version of the manuscript.
